# Neutrophil percentage-to-albumin ratio and all-cause mortality in cancer survivors with cardiovascular disease: Evidence from NHANES 1999–2018

**DOI:** 10.1097/MD.0000000000050768

**Published:** 2026-09-25

**Authors:** Lihua Lei, Yuanyuan Qin, Meiling Tang, Jianghuan Qin, Fangmei Wei, Qingqing Zeng

**Affiliations:** a The First Affiliated Hospital of Guilin Medical University, Guilin, Guangxi, China; b Department of Critical Care Medicine, The First Affiliated Hospital of Guangxi Medical University, Nanning, Guangxi, China; c Outpatient Department, The Second Affiliated Hospital of Guilin Medical University, Guilin, Guangxi, China; d Department of Burn and Plastic Surgery, The First Affiliated Hospital of Guangxi Medical University, Nanning, Guangxi, China; e Department of Otolaryngology, The Affiliated Wuming Hospital of Guangxi Medical University, Nanning, Guangxi, China; f Guangxi Medical University, Nanning, Guangxi, China.

**Keywords:** all-cause mortality, cancer survivors, cardiovascular disease, neutrophil percentage-to-albumin ratio, NHANES, prognostic biomarker

## Abstract

Neutrophil percentage-to-albumin ratio (NPAR) reflects both systemic inflammation and nutritional status, 2 factors closely linked to cancer progression and cardiovascular disease (CVD). While NPAR has been associated with adverse outcomes in various populations, its prognostic value in cancer survivors with comorbid CVD remains unclear. This study aimed to evaluate the association between NPAR and all-cause mortality in this high-risk population using nationally representative US data. We conducted a retrospective cohort study using data from the US National Health and Nutrition Examination Survey (NHANES, 1999–2018). A total of 1101 adult patients with both cancer and CVD were included. NPAR was evaluated as the exposure variable, and all-cause mortality was the outcome. Participants were categorized into tertiles according to NPAR. Kaplan–Meier survival curves were used to compare mortality across groups, and Cox proportional hazards regression models were applied to assess the independent association between NPAR and mortality risk. During a median follow-up of 77.9 months, higher NPAR levels were significantly associated with increased all-cause mortality. Kaplan–Meier analysis demonstrated significant differences in survival among tertiles (*P* < .0001). The median survival times were 73.0 months (IQR: 41.0–127.0), 70.0 months (IQR: 36.0–116.0), and 53.0 months (IQR: 26.8–102.0) for the lowest, middle, and highest tertiles, respectively. Multivariable Cox regression analysis confirmed that participants in the highest tertile had a significantly increased mortality risk compared with those in the middle tertile (HR = 1.54, 95% CI: 1.24–1.90, *P* < .001). Elevated NPAR is independently associated with higher all-cause mortality in adult cancer survivors with comorbid CVD. As a simple and readily available biomarker, NPAR may serve as a useful tool for early risk stratification and individualized management in this high-risk population.

## 1. Introduction

Advances in medical technology and treatment strategies have led to a steady increase in the global population of cancer survivors. According to projections by the American Cancer Society, approximately 20,41,910 new cancer cases and 6,18,120 cancer-related deaths are expected to occur in the United States in 2025.^[1]^ Despite improvements in oncological outcomes, cardiovascular disease (CVD) has emerged as a major comorbidity in this population. Large-scale epidemiological investigations indicate that nearly one-third of cancer patients develop CVD complications.^[2]^ Furthermore, the risk of CVD-related mortality among cancer survivors in the United States is significantly higher than in the general population, with several studies reporting a disproportionately elevated risk of CVD-related death in cancer patients compared to individuals without cancer.^[3]^

The neutrophil percentage-to-albumin ratio (NPAR) is a novel composite biomarker that integrates inflammatory and nutritional status, calculated as the ratio of neutrophil percentage to serum albumin concentration.^[4]^ This index provides a comprehensive reflection of systemic inflammation, mediated by neutrophils, and nutritional condition, represented by albumin levels.^[5]^ Both inflammation and malnutrition are well-recognized contributors to the onset and progression of cancer and cardiovascular disease, and they exert substantial influence on patient prognosis.^[6]^ Importantly, NPAR is easily obtainable, inexpensive, and simple to calculate, and previous studies have demonstrated its association with adverse outcomes across a wide range of conditions, including infectious diseases, diabetes, hypertension, heart failure, and cholelithiasis.^[7-11]^ Emerging evidence further suggests that NPAR may serve as a predictor of all-cause and cancer-specific mortality in oncology populations and is also linked to the risk and prevalence of cardiovascular disease.^[12,13]^

By simultaneously capturing inflammatory and nutritional axes, NPAR has been proposed as a useful adjunct in risk stratification and clinical decision-making. Recent investigations have shown that NPAR effectively predicts all-cause and disease-specific mortality in diverse populations, including those with chronic illnesses, critically ill patients, and even the general community.^[14,15]^ Findings from large-scale cohort studies, such as the United States (US) National Health and Nutrition Examination Survey (NHANES), further confirm that NPAR independently predicts both all-cause and cardiovascular mortality, underscoring its prognostic value.^[16]^ Nevertheless, the prognostic implications of NPAR in cancer survivors with concomitant cardiovascular disease remain insufficiently characterized. To date, systematic studies specifically addressing this high-risk population are scarce, and robust evidence to support the clinical application of NPAR in this context is lacking.

Based on these considerations, the present study employed a retrospective cohort design using large-scale data from NHANES 1999–2018 to systematically evaluate the association between NPAR and all-cause mortality among adult cancer patients with comorbid cardiovascular disease. Rigorous inclusion and exclusion criteria were applied to minimize potential confounding, and multivariable adjustments were performed to ensure the accuracy and reproducibility of the results. This study not only aims to further clarify the predictive value and applicability of NPAR in this high-risk population but also provides important evidence for the role of inflammation- and nutrition-related biomarkers in clinical risk stratification and individualized interventions. Ultimately, our findings may offer new perspectives for the management of patients with concurrent cancer and cardiovascular disease.

## 2. Materials and methods

### 2.1. Study population

This retrospective cohort study utilized data from NHANES spanning 1999 to 2018, with follow-up completed in 2018. The study population consisted of all adult cancer survivors identified in the NHANES database during this period. NHANES was conducted with approval by the National Center for Health Statistics Ethics Review Board and obtained informed written consent from all individuals involved in the study. A total of 5166 individuals were initially screened. Participants with missing albumin data (n = 736) or missing neutrophil percentage data (n = 22) were excluded. In addition, individuals without a history of cardiovascular disease (CVD) (n = 3307) were removed from the cohort. After application of these inclusion and exclusion criteria, 1101 adult cancer survivors with comorbid CVD remained eligible for analysis. The inclusion and exclusion process is illustrated in Figure 1. Disease diagnoses were defined according to NHANES standards, based on physician interviews, self-reported questionnaires, physical examinations, and laboratory testing. Data collection followed a structured and standardized protocol, and all procedures were performed by trained personnel to ensure accuracy and reliability.

**Figure 1.**
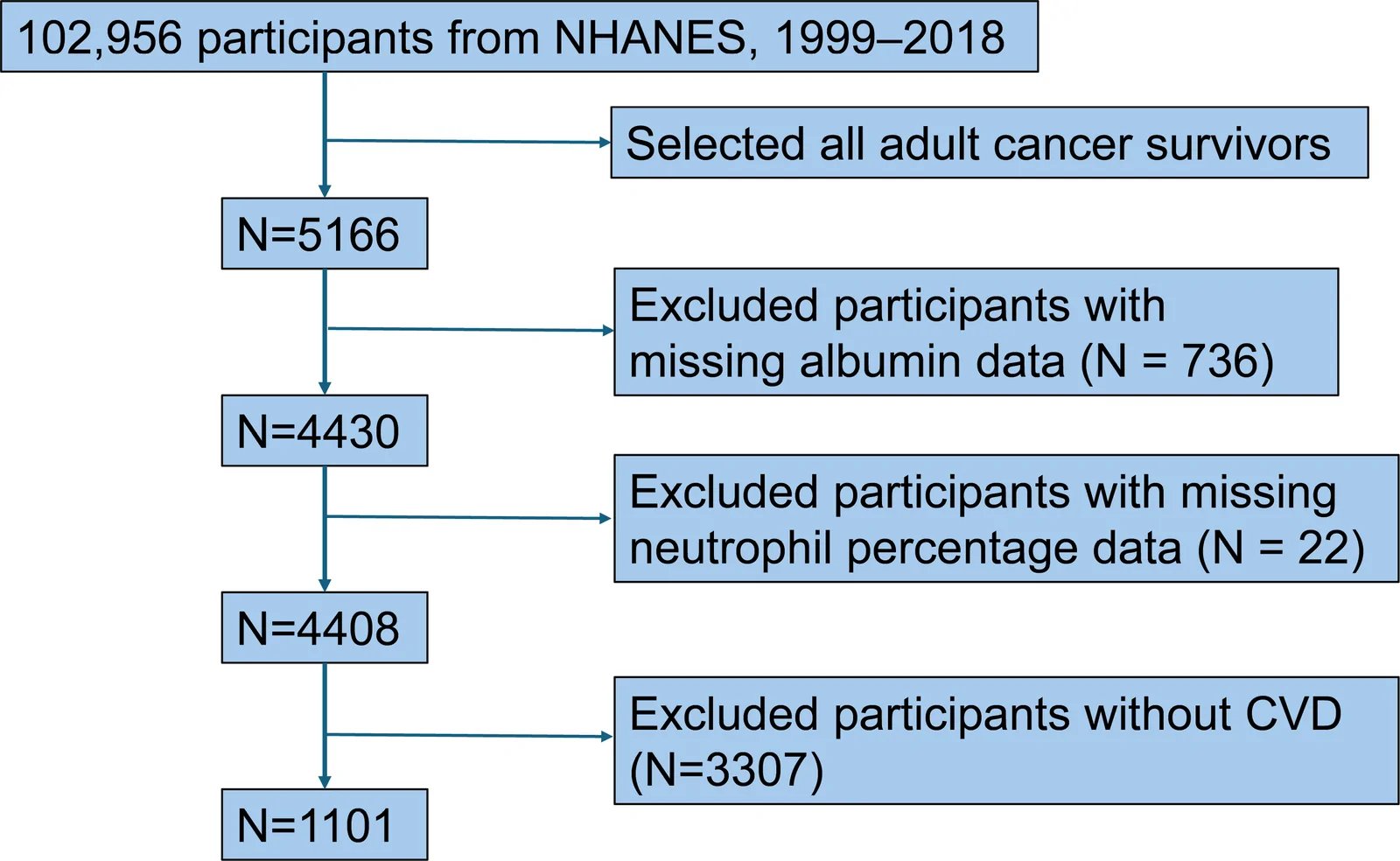
Flowchart of participant selection. CVD = cardiovascular disease, NHANES = National Health and Nutrition Examination Survey.

### 2.2. Study variables

The primary exposure variable was NPAR, calculated as neutrophil percentage multiplied by 100 and divided by albumin concentration (g/dL). All laboratory data were obtained from NHANES standardized test results. NPAR was analyzed both as a continuous variable and as a categorical variable stratified into tertiles to evaluate dose–response relationships. The primary outcome was all-cause mortality among cancer survivors with CVD, determined through linkage with the National Death Index. In accordance with previous epidemiological research, all-cause mortality was defined as death from any recorded cause.^[17]^

Covariates included demographic characteristics such as age, sex, race, marital status, education level, and poverty–income ratio; lifestyle factors including smoking and alcohol consumption; and clinical conditions such as hypertension, diabetes, hyperlipidemia, and pregnancy. Additional laboratory parameters included glycated hemoglobin, creatinine, total protein, uric acid, calcium, total cholesterol, triglycerides, white blood cell count, red blood cell count, platelet count, and lymphocyte count. These covariates were selected based on established epidemiological evidence in order to minimize residual confounding. All measurements and classifications adhered to NHANES standards.

### 2.3. Ethics statement

The study was conducted in accordance with the Declaration of Helsinki. The NHANES project was approved by the Institutional Review Board of the National Center for Health Statistics (NCHS) and the National Institutes of Health (NIH). Written informed consent was obtained from all participants at the time of enrollment. Because the present study was a secondary analysis of publicly available, de-identified data, no additional informed consent was required. All procedures related to data collection, storage, and use complied with international standards for data security and privacy protection.

### 2.4. Statistical analysis

Continuous variables with normal distributions are reported as mean ± standard deviation (SD), and comparisons between groups were assessed using Student *t* test or analysis of variance (ANOVA). Non-normally distributed continuous variables are expressed as median and interquartile range, with group differences tested using the Mann–Whitney *U* test or Kruskal–Wallis H test. Categorical variables are presented as frequencies and percentages, and differences between groups were examined using the χ^2^ test or Fisher exact test.

NPAR was categorized into tertiles for survival analyses. Kaplan–Meier curves with log-rank tests were used to compare all-cause mortality across groups. Associations between NPAR and mortality risk were evaluated using Cox proportional hazards regression models. A stepwise modeling approach was applied. The unadjusted model (model 0) was followed by successive adjustments. Model 1 adjusted for age and sex. Model 2 included additional adjustments for race, marital status, poverty–income ratio, and education level. Model 3 further incorporated smoking status, alcohol consumption, body mass index, diabetes, hyperlipidemia, and hypertension. Model 4 extended the adjustments to include laboratory biomarkers such as glycated hemoglobin, creatinine, total protein, uric acid, calcium, lipid profiles, and complete blood count parameters. Hazard ratios (HRs) and 95% confidence intervals (CIs) were reported.

To assess robustness, subgroup and sensitivity analyses were conducted. Missing data for covariates were handled by multiple imputation. All statistical analyses were performed using R statistical software (R Foundation for Statistical Computing, Vienna, Austria). Two-sided *P* values < .05 were considered statistically significant.

## 3. Results

### 3.1. Baseline characteristics of the study population

A total of 1101 adult cancer survivors with comorbid CVD were included in the analysis, of whom 56.8% were male and 43.2% were female. The mean age of the study population was 71.8 ± 11.1 years. Participants were stratified into tertiles (lowest NPAR tertile to highest NPAR tertile [Q1–Q3]) according to NPAR. Baseline characteristics are summarized in Table 1. Significant differences in demographic, lifestyle, and clinical biochemical indicators were observed across tertiles. Notably, higher NPAR levels were associated with older age, higher body mass index (BMI), and increased values of several laboratory parameters, whereas the proportions of married individuals and current alcohol consumers declined with rising NPAR levels (Table 1).

**Table 1:** Baseline characteristics by NPAR tertiles.

Variables	Total (n = 1101)	Q1 (n = 364)	Q2 (n = 369)	Q3 (n = 368)	*P* value
Age, mean ± SD	71.8 ± 11.1	69.6 ± 12.0	72.9 ± 9.9	72.8 ± 10.9	<.001
Sex, n (%)					.347
Male	625 (56.8)	196 (53.8)	218 (59.1)	211 (57.3)	
Female	476 (43.2)	168 (46.2)	151 (40.9)	157 (42.7)	
Race, n (%)					.071
Non-Hispanic White	818 (74.3)	249 (68.4)	280 (75.9)	289 (78.5)	
Non-Hispanic Black	146 (13.3)	61 (16.8)	45 (12.2)	40 (10.9)	
Mexican American	64 (5.8)	24 (6.6)	20 (5.4)	20 (5.4)	
Other Hispanic	32 (2.9)	16 (4.4)	11 (3)	5 (1.4)	
Other race – including multiracial	41 (3.7)	14 (3.8)	13 (3.5)	14 (3.8)	
Marry, n (%)					.008
Married/living with partner	605 (55.0)	214 (58.8)	213 (57.7)	178 (48.4)	
Never married/other	496 (45.0)	150 (41.2)	156 (42.3)	190 (51.6)	
PIR_group1, n (%)					.593
≤1.30	335 (30.4)	113 (31)	103 (27.9)	119 (32.3)	
1.31–3.50	495 (45.0)	156 (42.9)	175 (47.4)	164 (44.6)	
>3.50	271 (24.6)	95 (26.1)	91 (24.7)	85 (23.1)	
Education1, n (%)					.538
Less than high school	331 (30.1)	104 (28.6)	107 (29)	120 (32.6)	
High school or equivalent	252 (22.9)	81 (22.3)	93 (25.2)	78 (21.2)	
Above high school	518 (47.0)	179 (49.2)	169 (45.8)	170 (46.2)	
Smoke, n (%)					.152
Never	382 (34.7)	124 (34.1)	139 (37.7)	119 (32.3)	
Former	538 (48.9)	169 (46.4)	181 (49.1)	188 (51.1)	
Current	181 (16.4)	71 (19.5)	49 (13.3)	61 (16.6)	
Drink, n (%)					.016
Never	162 (14.7)	51 (14)	54 (14.6)	57 (15.5)	
Former	390 (35.4)	113 (31)	124 (33.6)	153 (41.6)	
Current	549 (49.9)	200 (54.9)	191 (51.8)	158 (42.9)	
BMI_kg/m^2^, mean ± SD	29.2 ± 6.3	28.6 ± 5.4	29.2 ± 6.4	29.8 ± 7.0	.024
DM, n (%)					.132
No	675 (61.3)	238 (65.4)	222 (60.2)	215 (58.4)	
Yes	426 (38.7)	126 (34.6)	147 (39.8)	153 (41.6)	
Hyperlipidemia, n (%)					.28
No	124 (11.3)	35 (9.6)	49 (13.3)	40 (10.9)	
Yes	977 (88.7)	329 (90.4)	320 (86.7)	328 (89.1)	
Hypertension, n (%)					.474
No	213 (19.3)	76 (20.9)	73 (19.8)	64 (17.4)	
Yes	888 (80.7)	288 (79.1)	296 (80.2)	304 (82.6)	
HbA1c,%, mean ± SD	6.1 ± 1.1	6.0 ± 1.1	6.0 ± 1.0	6.2 ± 1.2	.032
Cr, median (IQR) mg/dL	1.0 (0.8, 1.3)	1.0 (0.8, 1.2)	1.0 (0.8, 1.2)	1.1 (0.9, 1.4)	<.001
TP, mean ± SD g/dL	7.0 ± 0.5	7.2 ± 0.5	7.1 ± 0.5	6.9 ± 0.5	<.001
Ca, mg/dL, Mean ± SD	9.4 ± 0.4	9.5 ± 0.4	9.4 ± 0.4	9.3 ± 0.4	<.001
UA, mg/dL, mean ± SD	6.1 ± 1.7	6.0 ± 1.5	6.0 ± 1.7	6.3 ± 1.8	.039
TR, mg/dL, mean ± SD	181.5 ± 46.5	191.5 ± 48.4	178.5 ± 41.9	174.7 ± 47.4	<.001
TC, mg/dL, median (IQR)	135.0 (95.0, 201.0)	143.5 (100.8, 212.0)	134.0 (90.0, 192.0)	132.0 (93.8, 190.8)	.06
WBC, 10^3^ cells/µL, median (IQR)	7.1 (5.8, 8.6)	6.7 (5.4, 8.1)	6.9 (5.7, 8.3)	7.8 (6.4, 9.4)	<.001
RBC, 10^6^ cells/µL, mean ± SD	4.5 ± 0.6	4.5 ± 0.5	4.5 ± 0.5	4.4 ± 0.6	.009
PLT, 10^3^ cells/µL, mean ± SD	231.9 ± 73.9	231.8 ± 70.2	233.6 ± 70.9	230.2 ± 80.2	.823
LC, 10^3^ cells/µL, median (IQR)	1.7 (1.3, 2.3)	2.2 (1.7, 2.8)	1.7 (1.3, 2.1)	1.4 (1.1, 1.8)	<.001
Time_exm, median (IQR)	67.0 (33.0, 113.0)	73.0 (41.0, 127.0)	70.0 (36.0, 116.0)	53.0 (26.8, 102.0)	<.001

Values are presented as mean ± standard deviation (SD), median with interquartile range (IQR), or number (percentage), as appropriate. Differences among groups were assessed using 1-way ANOVA or Kruskal–Wallis test for continuous variables and the χ^2^ test for categorical variables.

BMI = body mass index, Ca = calcium, Cr = creatinine, DM = diabetes mellitus, IQR = interquartile range, LC = lymphocyte count, NPAR = neutrophil percentage-to-albumin ratio, PIR = poverty income ratio, PLT = platelet count, RBC = red blood cell count, SD = standard deviation, TC = total cholesterol, Time_exm = follow-up time (months), TP = total protein, TR = triglycerides, UA = uric acid, WBC = white blood cell count.

### 3.2. Multivariable Cox regression

Multivariable Cox proportional hazards regression demonstrated a significant association between elevated NPAR and increased risk of all-cause mortality. Compared with the intermediate tertile (Q2), participants in the highest tertile (Q3) had a markedly increased risk of death, and this relationship persisted after adjustment for multiple covariates (Table 1). Trend analysis indicated a positive association between increasing NPAR and mortality risk. In the fully adjusted model (model 4), each increment in NPAR was associated with a significantly higher mortality risk (HR = 1.28, 95% CI: 1.13–1.45, *P* < .001). Detailed results are shown in Table 2.

**Table 2:** Cox regression analysis of NPAR and all-cause mortality across different models.

Group	Model 0		Model 1		Model 2		Model 3		Model 4	
	HR (95% CI)	*P*	HR (95% CI)	*P*	HR (95% CI)	*P*	HR (95% CI)	*P*	HR (95% CI)	*P*
NPAR	3.02 (2.26–4.04)	<.001	2.56 (1.9–3.46)	<.001	2.38 (1.76–3.23)	<.001	2.4 (1.76–3.26)	<.001	2.34 (1.58–3.45)	<.001
Q2	1		1		1		1		1	
Q1	0.77 (0.62–0.95)	.015	0.91 (0.73–1.13)	.383	0.93 (0.75–1.16)	.518	0.93 (0.75–1.16)	.527	0.96 (0.76–1.21)	.722
Q3	1.56 (1.29–1.88)	<.001	1.59 (1.31–1.92)	<.001	1.54 (1.27–1.87)	<.001	1.57 (1.29–1.91)	<.001	1.54 (1.24–1.9)	<.001
Trend.test	1.43 (1.29–1.59)	<.001	1.34 (1.21–1.49)	<.001	1.3 (1.17–1.45)	<.001	1.32 (1.19–1.47)	<.001	1.28 (1.13–1.45)	<.001

Hazard ratios (HRs) with 95% confidence intervals (CIs) were calculated using Cox proportional hazards regression models. Model 0 was unadjusted; model 1 adjusted for age and sex; model 2 additionally adjusted for race/ethnicity, marital status, poverty income ratio (PIR), and education; model 3 further adjusted for smoking status, alcohol consumption, body mass index (BMI), diabetes mellitus, hyperlipidemia, and hypertension; Model 4 additionally adjusted for HbA1c, creatinine, total protein, uric acid, calcium, total cholesterol, triglycerides, white blood cell count, red blood cell count, platelet count, and lymphocyte count.

CI = confidence interval, HR = hazard ratio, NPAR = neutrophil percentage-to-albumin ratio.

### 3.3. Survival analysis

Kaplan–Meier survival analysis revealed significant differences in all-cause survival across the 3 NPAR groups (*P* < .0001). The mean survival time was 85.6 months in Q1, 80.8 months in Q2 (intermediate NPAR tertile), and 67.2 months in Q3. Over the follow-up period, survival probability in the highest tertile remained consistently lower than in the middle and lowest tertiles, while participants in Q1 exhibited the most favorable survival outcomes. The numbers at risk across groups were comparable at baseline and declined proportionally over time (Fig. 2).

**Figure 2.**
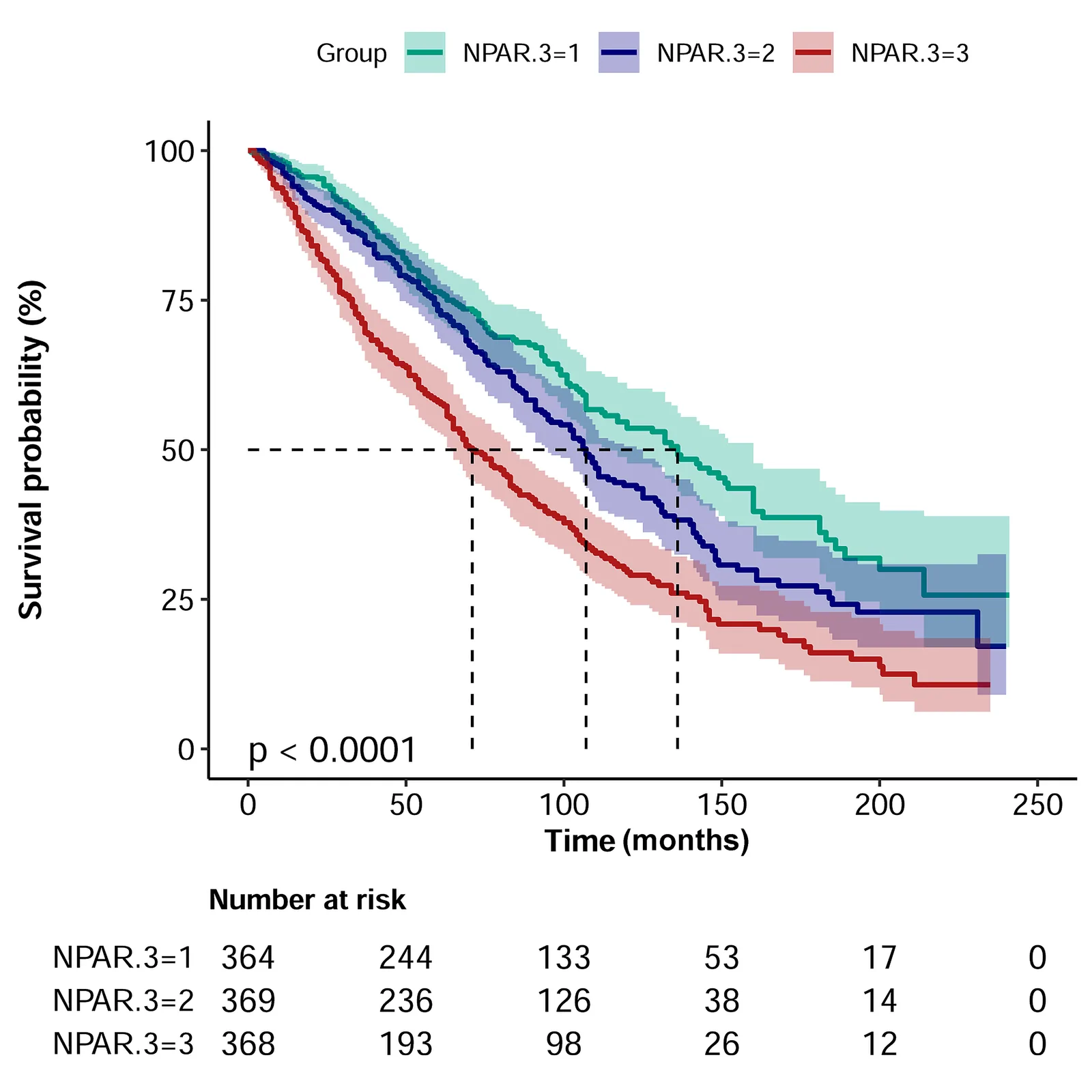
Kaplan–Meier curves for all-cause mortality according to NPAR tertiles. NPAR = neutrophil percentage-to-albumin ratio.

### 3.4. Nonlinear relationship

Cox spline and threshold effect analyses were conducted to explore the shape of the association between NPAR and mortality (Table 3 and Fig. 3). The results indicated a nonlinear relationship. A threshold effect was observed at an NPAR value of 1.209. Above this threshold, mortality risk increased significantly with higher NPAR, whereas changes below this point were not statistically meaningful. The overall association was highly significant (*P* overall < .001), with strong evidence of nonlinearity (*P* for nonlinearity < .001).

**Table 3:** Association between NPAR and all-cause mortality: Cox breakpoint and nonlinearity analysis.

Item	Breakpoint.HR (95% CI)	*P* value
E_BK1	1.209 (1.179, 1.238)	NA
Slope1	0.185 (0.02, 1.735)	.1396
Slope2	2.804 (1.61, 4.885)	<.001
Likelihood ratio test	–	.011
Nonlinear Test*1	–	.004
Nonlinear Test*2	–	.005

Breakpoint and slope estimates were derived from Cox proportional hazards regression models with segmented (piecewise) analysis. The likelihood ratio test was used to compare the segmented model with a single linear model. Nonlinear associations between NPAR and all-cause mortality were evaluated using restricted cubic spline functions (*1 and *2 denote different spline model specifications).

CI = confidence interval, HR = hazard ratio, NA = not applicable, NPAR = neutrophil percentage-to-albumin ratio.

**Figure 3.**
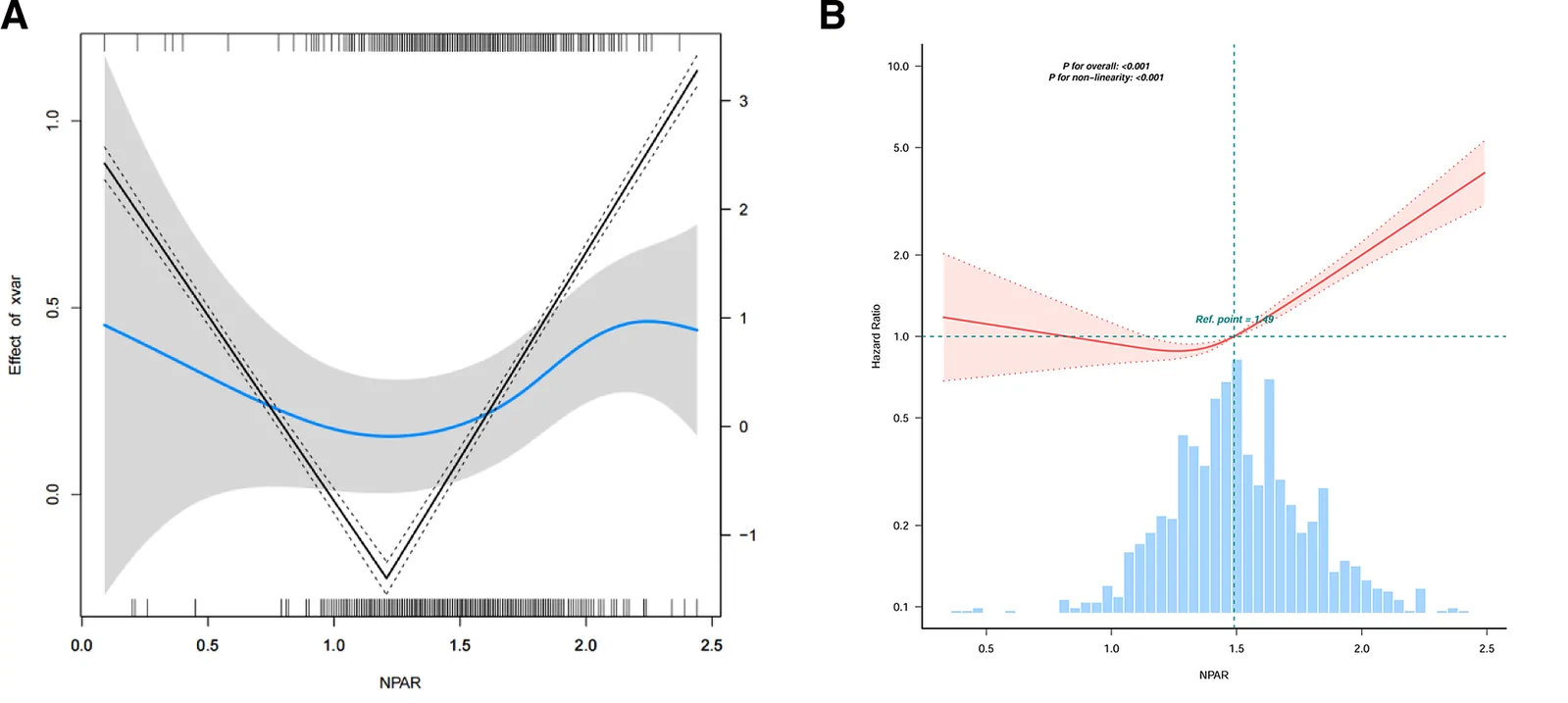
Nonlinear relationship between neutrophil-to-albumin ratio (NPAR) and all-cause mortality risk. (A) Piecewise Cox regression (threshold) analysis (B) Smooth curve fitting (Cox model). NPAR = neutrophil percentage-to-albumin ratio.

### 3.5. ROC curve analysis

Receiver operating characteristic (ROC) curve analysis yielded an area under the curve (AUC) of 0.602 (95% CI: 0.568–0.635), indicating limited discriminatory capacity (Fig. 4). The optimal cutoff value for NPAR was 1.545, corresponding to a sensitivity of 0.669 and a specificity of 0.492.

**Figure 4.**
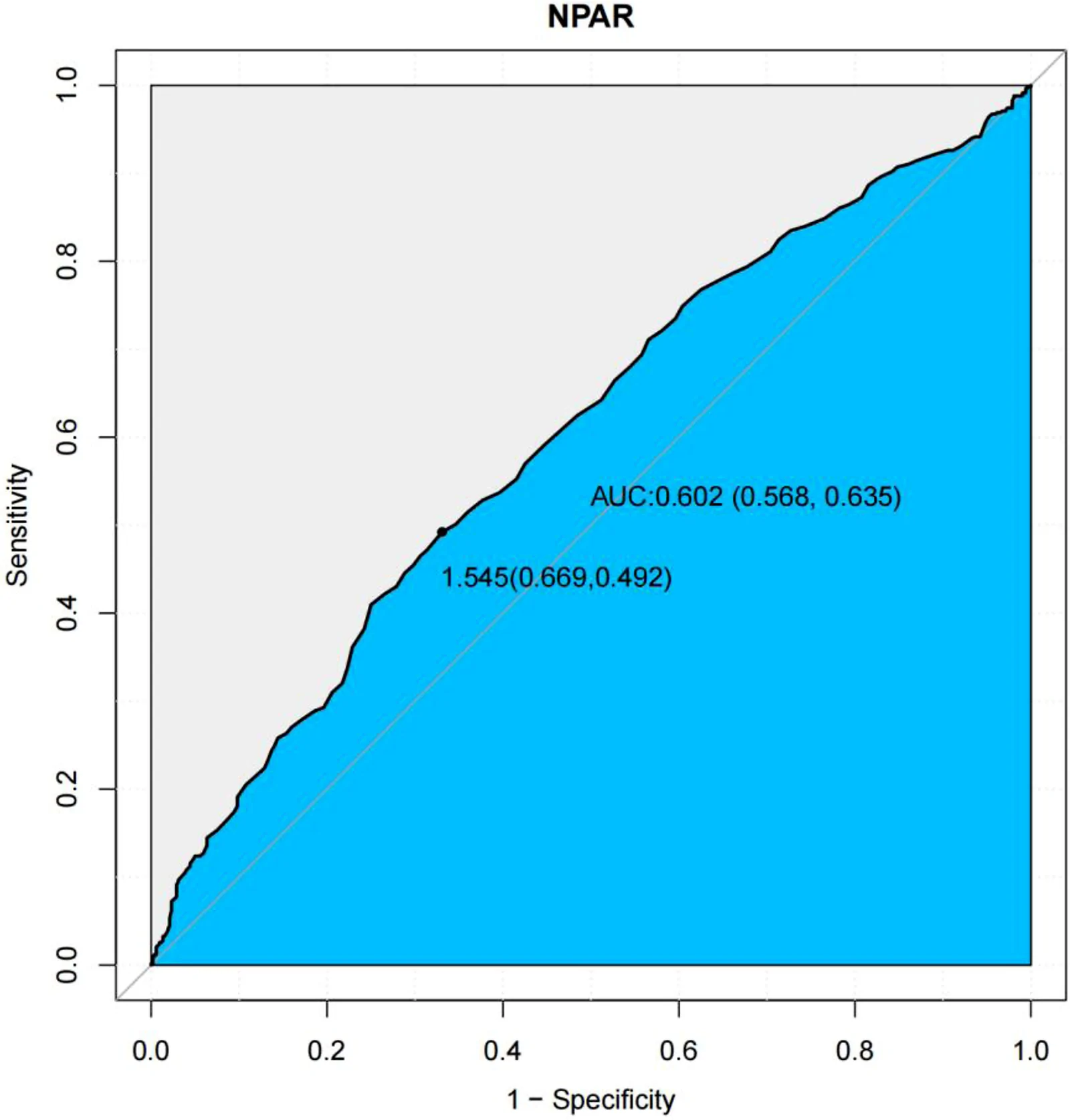
ROC curve of NPAR. AUC = area under the curve, NPAR = neutrophil percentage-to-albumin ratio, ROC = receiver operating characteristic.

### 3.6. Subgroup analysis

Subgroup analyses stratified by sex, age, marital status, income, education, smoking and alcohol status, chronic comorbidities, and BMI consistently demonstrated that higher NPAR was associated with increased all-cause mortality risk. For example, among men, elevated NPAR was significantly associated with mortality (adjusted HR = 2.15, 95% CI: 1.27–3.64), and a similar association was observed among women (adjusted HR = 2.42, 95% CI: 1.20–4.87). No significant interactions were observed between NPAR and any stratification variable (all *P* for interaction > 0.05), indicating the robustness of the association across different population subgroups. The detailed results are presented in Table 4 and Figure 5.

**Table 4:** Subgroup analyses of the association between neutrophil-to-albumin ratio (NPAR) and all-cause mortality.

Subgroup	n (total)	n (events) (%)	Crude HR (95% CI)	Crude *P*-value	Adjusted HR (95% CI)	Adjusted *P*-value	*P* for interaction
Sex							
Male	625	345 (55.2)	2.83 (1.91–4.19)	<.001	2.15 (1.27–3.64)	.005	.446
Female	476	236 (49.6)	3.24 (2.09–5.03)	<.001	2.42 (1.20–4.87)	.013	
Age (yr)							
<70	355	109 (30.7)	3.59 (1.84–6.98)	<.001	2.60 (0.79–8.55)	.117	.275
≥70	746	472 (63.3)	2.44 (1.75–3.40)	<.001	2.19 (1.41–3.41)	<.001	
Marital status							
Married/partnered	605	299 (49.4)	2.56 (1.66–3.95)	<.001	2.89 (1.50–5.58)	.002	.144
Never/other	496	282 (56.9)	3.28 (2.20–4.89)	<.001	2.80 (1.58–4.94)	<.001	
PIR							
≤1.30	335	174 (51.9)	3.40 (1.97–5.87)	<.001	3.89 (1.41–10.74)	.009	.426
1.31–3.50	495	287 (58.0)	3.06 (2.04–4.60)	<.001	2.12 (1.21–3.73)	.009	
>3.50	271	120 (44.3)	2.46 (1.27–4.78)	.008	2.66 (1.01–7.00)	.048	
Education							
<High school	331	206 (62.2)	2.01 (1.26–3.21)	.003	1.80 (0.87–3.74)	.114	.272
High school	252	127 (50.4)	3.87 (2.00–7.50)	<.001	4.52 (1.81–11.32)	.001	
>High school	518	248 (47.9)	3.84 (2.41–6.10)	<.001	3.03 (1.56–5.90)	.001	
Smoking							
Never	382	192 (50.3)	2.37 (1.41–3.99)	.001	2.29 (1.16–4.52)	.017	.997
Former	538	309 (57.4)	3.50 (2.34–5.24)	<.001	2.32 (1.30–4.16)	.005	
Current	181	80 (44.2)	2.97 (1.41–6.25)	.004	2.75 (0.55–13.78)	.219	
Drinking							
Never	162	91 (56.2)	2.37 (1.15–4.89)	.019	3.51 (1.07–11.53)	.039	.969
Former	390	239 (61.3)	2.68 (1.74–4.12)	<.001	2.35 (1.16–4.73)	.017	
Current	549	251 (45.7)	3.27 (2.03–5.26)	<.001	2.72 (1.43–5.19)	.002	
Diabetes							
No	675	355 (52.6)	2.64 (1.82–3.83)	<.001	2.25 (1.35–3.76)	.002	.422
Yes	426	226 (53.1)	3.83 (2.41–6.09)	<.001	2.93 (1.44–5.96)	.003	
Hyperlipidemia							
No	124	76 (61.3)	2.28 (0.87–5.98)	.093	27.26 (2.87–258.88)	.004	.253
Yes	977	505 (51.7)	3.09 (2.27–4.20)	<.001	2.41 (1.59–3.65)	<.001	
Hypertension							
No	213	110 (51.6)	3.23 (1.69–6.15)	<.001	2.44 (0.76–7.90)	.135	.657
Yes	888	471 (53.0)	2.97 (2.14–4.11)	<.001	2.70 (1.74–4.20)	<.001	
BMI (kg/m^2^)							
<25	268	174 (64.9)	3.48 (1.87–6.46)	<.001	2.34 (0.98–5.56)	.055	.696
25–29.9	409	218 (53.3)	2.53 (1.59–4.02)	<.001	2.12 (1.06–4.24)	.034	
≥30	424	189 (44.6)	3.83 (2.34–6.28)	<.001	3.62 (1.77–7.40)	<.001	

Hazard ratios (HRs) with 95% confidence intervals (CIs) were estimated using Cox proportional hazards regression models. Crude HRs represent unadjusted associations, while adjusted HRs were derived from multivariable models controlling for age, sex, race/ethnicity, marital status, poverty income ratio (PIR), education, smoking status, alcohol consumption, body mass index (BMI), diabetes mellitus, hyperlipidemia, hypertension, HbA1c, creatinine, total protein, uric acid, calcium, total cholesterol, triglycerides, white blood cell count, red blood cell count, platelet count, and lymphocyte count. P for interaction tests were conducted to evaluate potential effect modification across subgroups.

BMI = body mass index, CI = confidence interval, HR = hazard ratio, NPAR = neutrophil percentage-to-albumin ratio, PIR = poverty income ratio.

**Figure 5.**
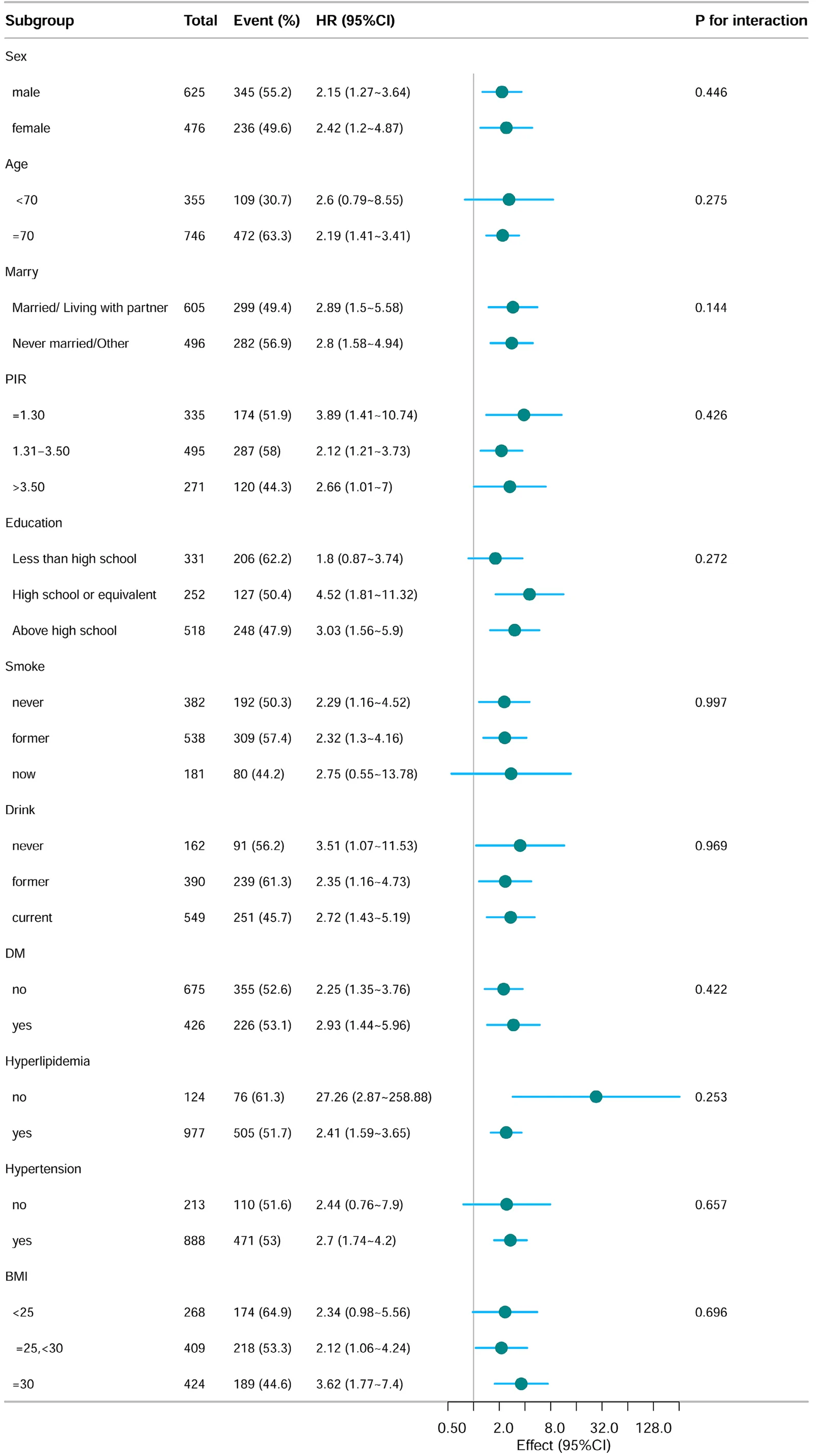
Subgroup analyses of NPAR and all-cause mortality. BMI = body mass index, CI = confidence interval, DM = diabetes mellitus, HR = hazard ratio, NPAR = neutrophil percentage-to-albumin ratio, PIR = poverty income ratio.

## 4. Discussion

In this large, nationally representative retrospective cohort study based on NHANES 1999–2018 with long-term follow-up, we systematically examined the association between NPAR and all-cause mortality among adult cancer survivors with comorbid CVD. Our findings demonstrate that elevated NPAR was significantly associated with an increased risk of all-cause mortality. Even after adjusting for demographic, socioeconomic, lifestyle, and clinical covariates, participants in the highest NPAR tertile had a markedly higher risk of death compared with those in the intermediate tertile (HR = 1.54, 95% CI: 1.24–1.90). Moreover, NPAR levels were positively correlated with mortality risk in a dose–response manner (*P* < .001). These results provide robust epidemiological evidence supporting the value of NPAR as an inflammatory–nutritional biomarker for risk stratification in this high-risk population.

Our results align with previous studies highlighting the prognostic role of NPAR in diverse populations. Prior work has demonstrated that higher NPAR values are associated with a greater prevalence of CVD in the general population and with increased all-cause and cancer-specific mortality in oncology cohorts.^[12,13]^ Similarly, Xie et al (2021) reported that elevated NPAR was associated with increased all-cause mortality in the general US population (HR = 1.31, 95% CI: 1.09–1.58).^[18]^ Compared with these studies, the present investigation is distinguished by its focus on cancer survivors with comorbid CVD, thus addressing a clinically important yet underexplored subgroup.

Consistent with our findings, Chen et al (2022) also demonstrated a positive association between NPAR and short- and long-term mortality among stroke patients using the Medical Information Mart for Intensive Care III (MIMIC-III) database, although their study was limited by a smaller sample size (n = 940) and shorter follow-up.^[19]^ In contrast, our study leveraged a larger, more representative cohort, incorporated a longer follow-up period, and employed more comprehensive covariate adjustments. Together, these differences underscore the robustness and clinical relevance of our findings.

The ROC analysis further informs the clinical interpretation of these findings. Although NPAR was independently associated with mortality in regression models, its AUC of 0.602 indicates limited discrimination when used alone. Thus, the principal value of NPAR may lie in its role as an inexpensive adjunct to established clinical risk factors rather than as an isolated screening or decision-making tool. Future studies should determine whether adding NPAR to validated prognostic models produces meaningful improvements in discrimination, calibration, and clinical utility.

From a mechanistic perspective, NPAR reflects 2 interrelated biological processes: systemic inflammation and nutritional status. Neutrophil percentage reflects the degree of immune activation and inflammatory response,^[20]^ while serum albumin is a well-established indicator of both nutritional status and systemic inflammation.^[21]^ Inflammatory states are associated with suppressed albumin synthesis, contributing to malnutrition and disease progression. By integrating these 2 markers, NPAR provides a more accurate measure of the inflammation–nutrition axis than either parameter alone. In cancer survivors, chronic tumor-associated inflammation and CVD-related immune activation coexist, creating a physiological environment in which NPAR can effectively capture both persistent inflammation and nutritional deterioration.^[22-25]^ These mechanisms likely explain why NPAR demonstrated independent prognostic value in this high-risk population.

The clinical burden experienced by patients with cancer and cardiovascular disease may also extend beyond mortality and include cognitive and functional consequences. Previous studies have emphasized the value of neuropsychological assessment for detecting cognitive deficits after cardiac surgery, described post-chemotherapy cognitive impairment across cancer types, and reported persistent postoperative cognitive dysfunction in older surgical populations.^[26-28]^ Although these outcomes were not assessed in NHANES and fall outside the primary scope of the present analysis, they further illustrate the multidimensional vulnerability of patients with cancer and cardiovascular comorbidity and support comprehensive follow-up beyond conventional survival outcomes.

The present study has several strengths. First, it is based on NHANES, a large and nationally representative dataset with long-term follow-up, thereby enhancing the generalizability and scientific validity of the findings. Second, the study population was specifically restricted to cancer survivors with CVD, which allowed us to address an important comorbidity context and minimized confounding from unrelated baseline diseases. Third, standardized definitions of exposure (NPAR) and outcome (all-cause mortality) ensured accuracy and comparability. Finally, the use of multivariable Cox regression, stepwise adjustment models, and both sensitivity and subgroup analyses strengthened the robustness of the conclusions and confirmed the consistency of results across diverse subgroups.

Despite these strengths, several limitations should be acknowledged. First, cancer and CVD diagnoses were based primarily on self-reported physician diagnoses, and information on disease stage, activity, treatment response, and recurrence was unavailable. Second, NHANES did not provide a single harmonized indicator across all 1999–2018 cycles for acute infection at the time of blood collection, recent blood transfusion, or specific myeloproliferative neoplasms. Prescription medication files recorded many medications used during the preceding 30 days but may not have comprehensively captured inpatient, infusion, short-course, or unreported immunosuppressive and antineoplastic treatments. These factors therefore could not be applied as uniform exclusion criteria and remain potential sources of residual confounding because they may affect neutrophil percentage, albumin concentration, and other complete blood count parameters. Third, cancer-specific subgroup analyses were not performed because several cancer categories contained small numbers of participants and deaths, which could have produced unstable estimates and increased the risk of chance findings arising from multiple comparisons. Moreover, cancer type, stage, activity, and treatment status were not sufficiently detailed for robust cancer-specific analyses; therefore, heterogeneity among cancer survivors may remain. Fourth, the prespecified endpoint was all-cause mortality. Although the public-use linked mortality files provide broad underlying cause-of-death categories, cardiovascular and noncardiovascular mortality were not analyzed separately. Accordingly, the present findings should not be interpreted as evidence that NPAR has equivalent associations with cardiovascular and other causes of death. Fifth, NHANES contains age-at-diagnosis information for selected cancer and cardiovascular conditions, allowing approximate disease duration to be calculated for some participants. However, availability and completeness vary according to disease and survey cycle, particularly among participants reporting multiple cancers or cardiovascular diagnoses. Disease duration therefore could not be consistently determined across the cohort and was not included as a covariate. Sixth, because this was an observational analysis, causal inference cannot be established. Finally, the cohort represents the noninstitutionalized US population, and external validation is required before extrapolating the findings to other settings or using NPAR in clinical decision-making. These limitations, common to studies using NHANES data,^[29]^ highlight the need for cautious interpretation and underscore the importance of validation through prospective, multicenter studies across diverse populations.

## 5. Conclusion

This study demonstrates that NPAR, an easily obtainable and cost-effective biomarker reflecting both systemic inflammation and nutritional status, is an independent predictor of all-cause mortality among adult cancer survivors with comorbid CVD. By facilitating early identification of high-risk individuals, NPAR may provide valuable support for clinical risk stratification and individualized management. Incorporating NPAR into the routine follow-up of cancer–CVD comorbidity patients could help optimize interventions, improve survival outcomes, and enhance quality of life. Future prospective studies integrating multi-omics approaches and dynamic monitoring are warranted to further validate its clinical utility and elucidate underlying biological mechanisms.

## Author contributions

**Conceptualization:** Lihua Lei, Qingqing Zeng.

**Data curation:** Lihua Lei, Yuanyuan Qin, Qingqing Zeng.

**Formal analysis:** Lihua Lei, Yuanyuan Qin, Meiling Tang, Jianghuan Qin, Qingqing Zeng.

**Funding acquisition:** Lihua Lei.

**Investigation:** Yuanyuan Qin, Meiling Tang, Fangmei Wei.

**Methodology:** Meiling Tang.

**Project administration:** Fangmei Wei.

**Resources:** Fangmei Wei.

**Software:** Meiling Tang, Jianghuan Qin.

**Validation:** Jianghuan Qin.

**Writing – original draft:** Lihua Lei, Yuanyuan Qin, Meiling Tang, Jianghuan Qin, Fangmei Wei, Qingqing Zeng.

**Writing – review & editing:** Lihua Lei, Yuanyuan Qin, Meiling Tang, Jianghuan Qin, Fangmei Wei, Qingqing Zeng.
